# Supplementary material for: In Vitro and In Silico Studies on Cytotoxic Properties of Oxythiamine and 2′-Methylthiamine
Source: Int J Mol Sci. 2024 Apr 15;25(8):4359. doi: 10.3390/ijms25084359 (PMC11050282; doi:10.3390/ijms25084359)
Supplement: Supplementary file 1 [file ijms-25-04359-s001.zip › ijms-2912255-supplementary.pdf]

SUPPORTING INFORMATION

In vitro and *in silico* studies on cytotoxic properties of oxythiamine and 2'-methylthiamine

Marta Malinowska[1,†], Magdalena Czerniecka[2,3†,*], Izabella Jastrzebska[1], Artur Ratkiewicz[1,*], Adam Tylicki[2] and Natalia Wawrusiewicz-Kurylonek[4]

[1] Faculty of Chemistry, University of Bialystok, Bialystok, Poland
[2] Faculty of Biology, University of Bialystok, Bialystok, Poland
[3] Laboratory of Tissue Culture, Department of Biology, University of Bialystok, Ciolkowskiego 1J, 15-245 Bialystok, Poland
[4] Department of Clinical Genetics, Medical University of Białystok, Waszyngtona 13, 15-089 Białystok, Poland
† These authors contributed equally to this work
* *Correspondence: m.siemieniuk@uwb.edu.pl (M.C.), artrat@uwb.edu.pl (A. R.)*

Copies of NMR spectra of new compounds

S1 – ^{1}H NMR spectrum of *4- Amino-2-methylpyrimidine- 5-carbonitrile (1)*………………………………………………………………… **3**

S2 – ^{13}C NMR spectrum of *4- Amino-2-methylpyrimidine- 5-carbonitrile (1)*………………………………………………………………… **4**

S3 – ^{1}H NMR spectrum of *4-Amino-2-methylpyrimidine-5-carboxaldehyde (2)*…………………………………………………… **5**

S4 – ^{13}C NMR spectrum of *4-Amino-2-methylpyrimidine-5-carboxaldehyde (2)*…………………………………………………… **6**

S5 – ^{1}H NMR spectrum of *(4-amino-2-methylpyrimidin-5-yl)methanol (3)*………………………………………………………… **7**

S6 – ^{13}C NMR spectrum of *(4-amino-2-methylpyrimidin-5-yl)methanol (3)*………………………………………………………… **8**

S7 – ^{1}H NMR spectrum of *2'-methylthiamine*……………………………………………………………………………………… **9**

S8 – ^{13}C NMR spectrum of *2'-methylthiamine*……………………………………………………………………………………… **10**

S9 – HPLC chromatogram of *2'-methylthiamine* ………………………………………………………………………………… **11**

S10 – ESI-HRMS of *2'-methylthiamine* …………………………………………………………………………………………… **12**

Table S1……… **13**

S1 – ^{1}H NMR spectrum of *4- Amino-2-methylpyrimidine- 5-carbonitrile (1)*

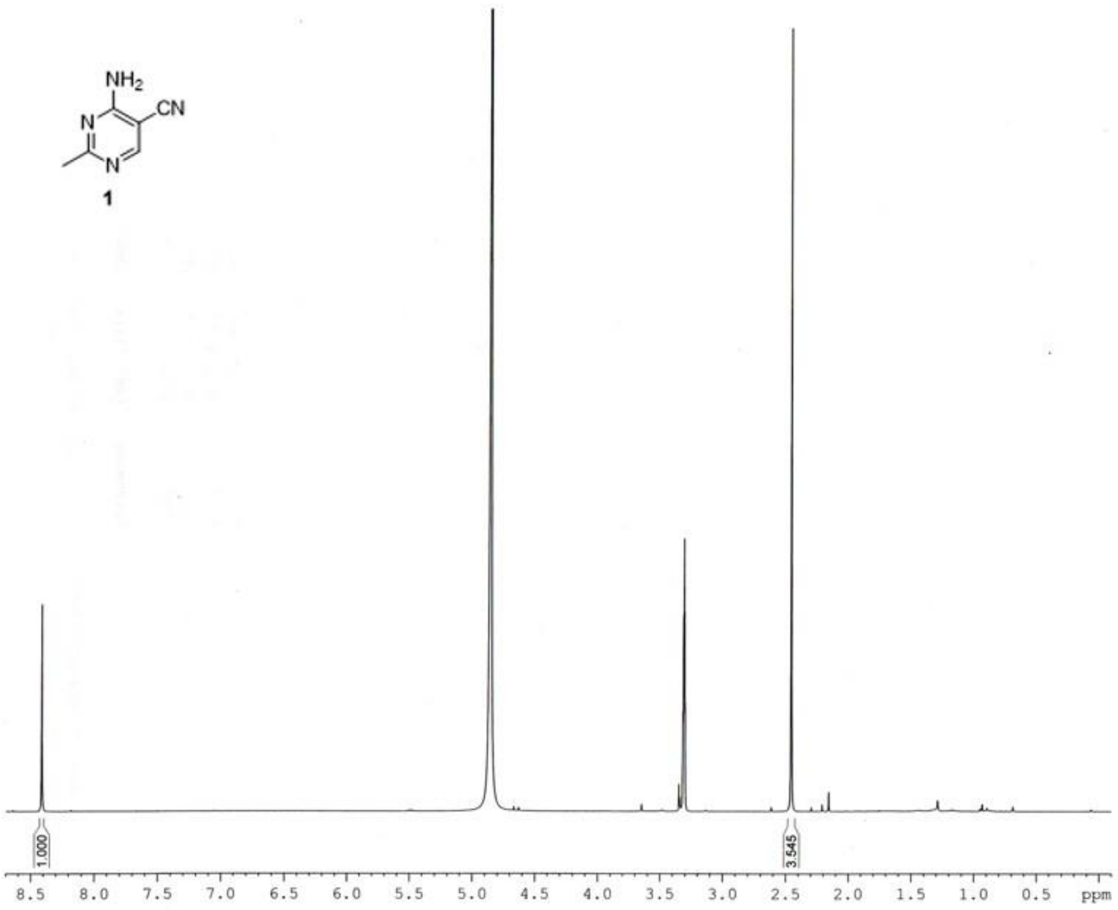

S2 – ^{13}C NMR spectrum of *4- Amino-2-methylpyrimidine- 5-carbonitrile (1)*

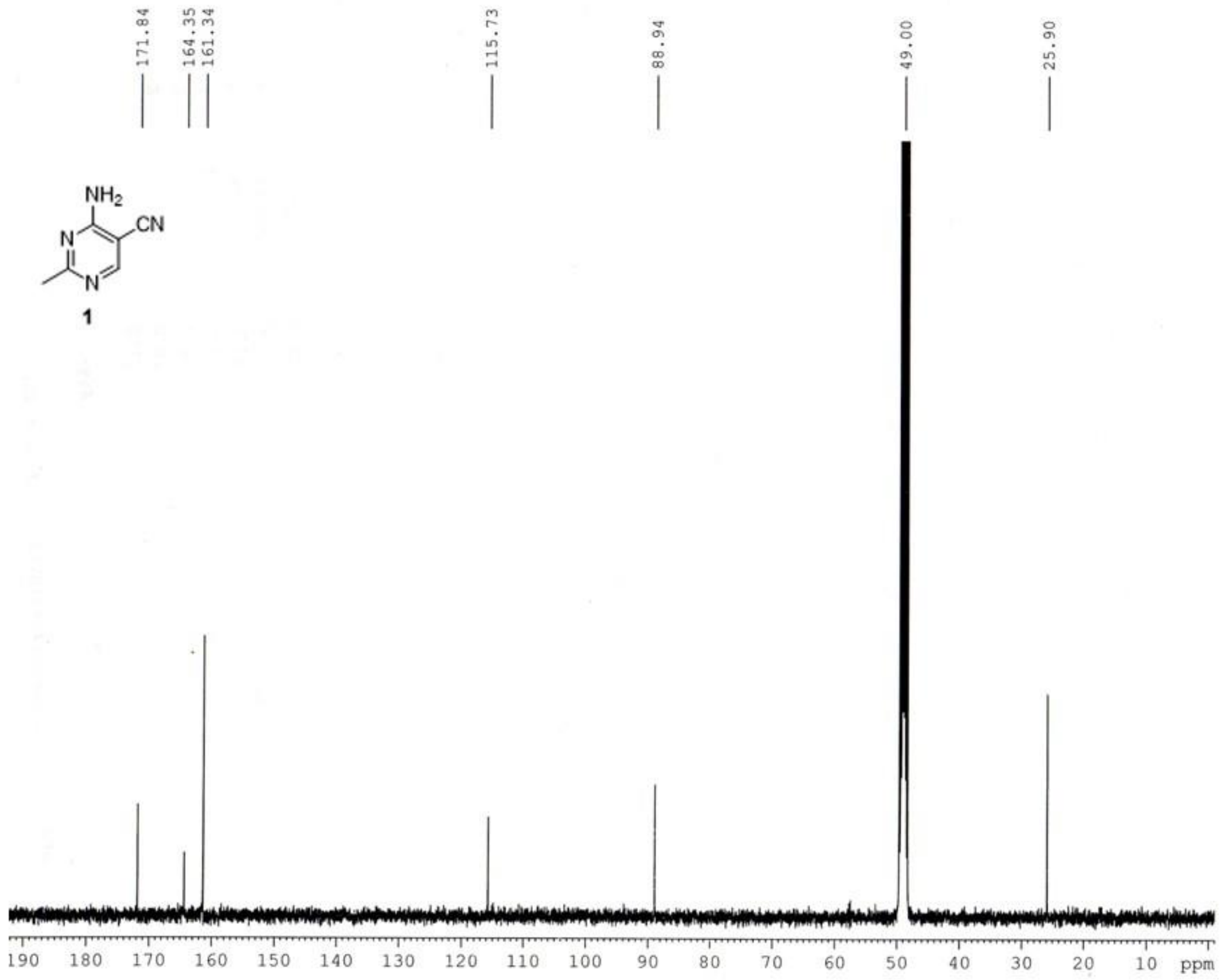

S3 – ^{1}H NMR spectrum of *4-Amino-2-methylpyrimidine-5-carboxaldehyde (2)*

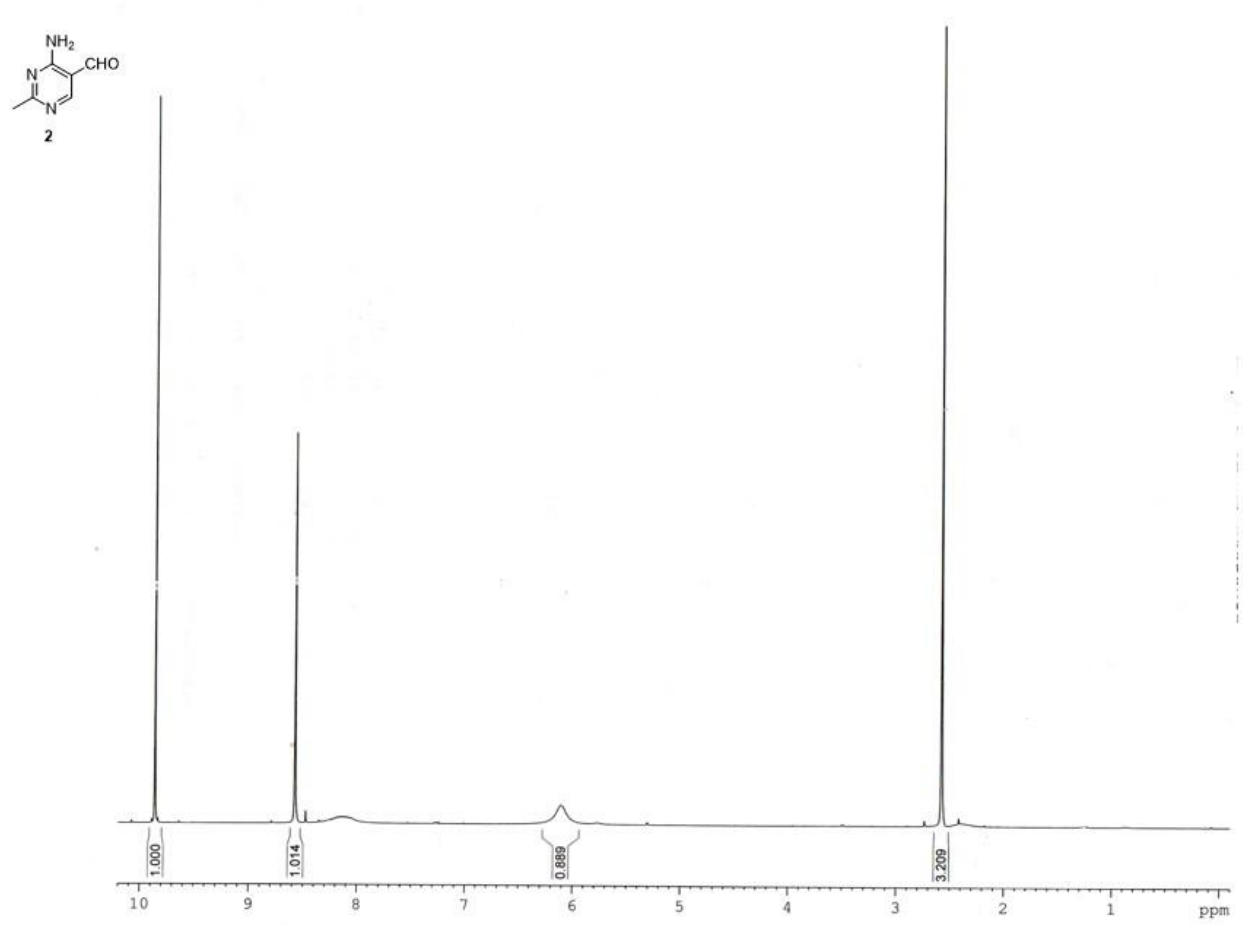

S4 – ^{13}C NMR spectrum of *4-Amino-2-methylpyrimidine-5-carboxaldehyde (2)*

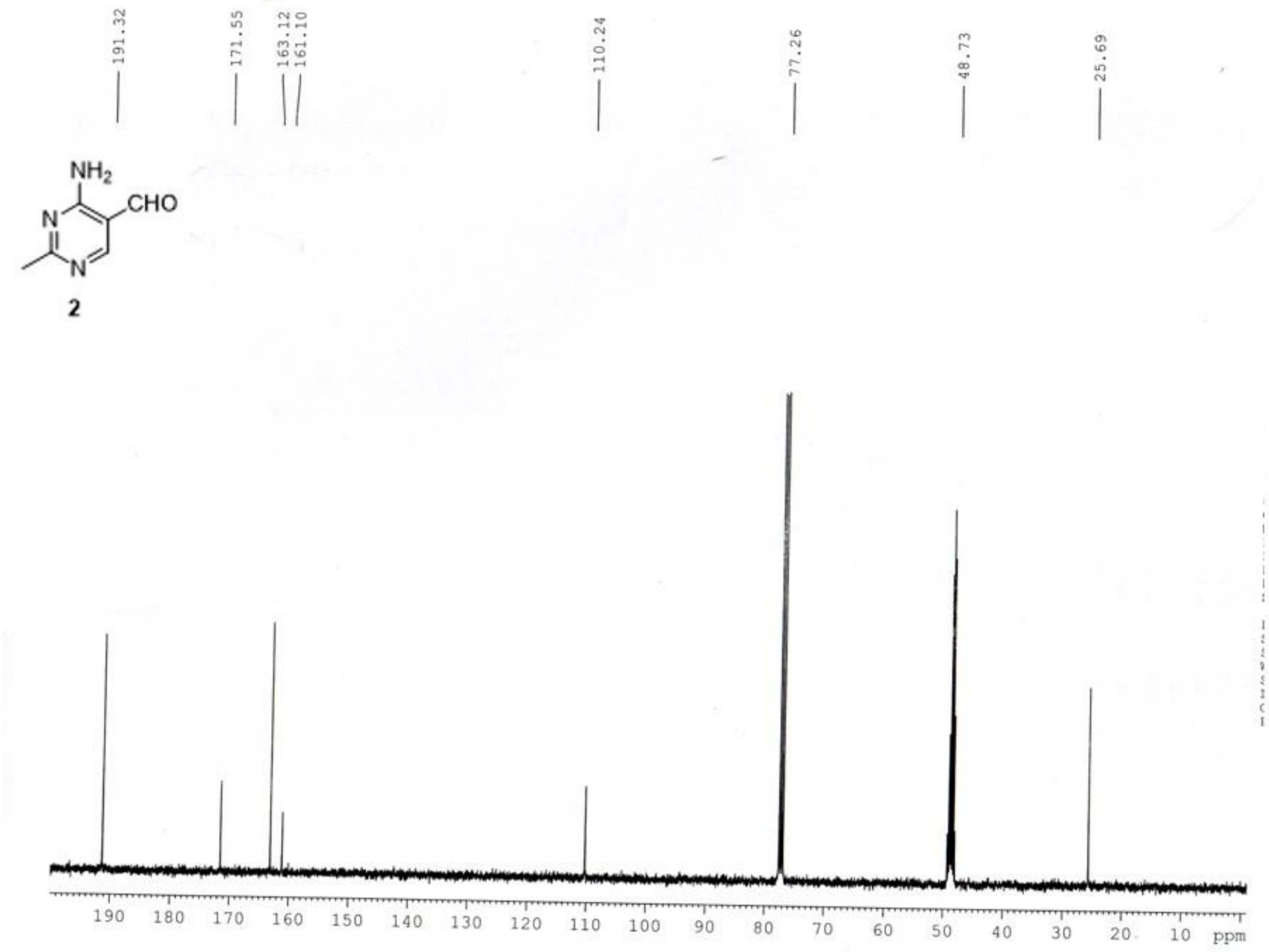

S5 – ^{1}H NMR spectrum of *(4-amino-2-methylpyrimidin-5-yl)methanol (3)*

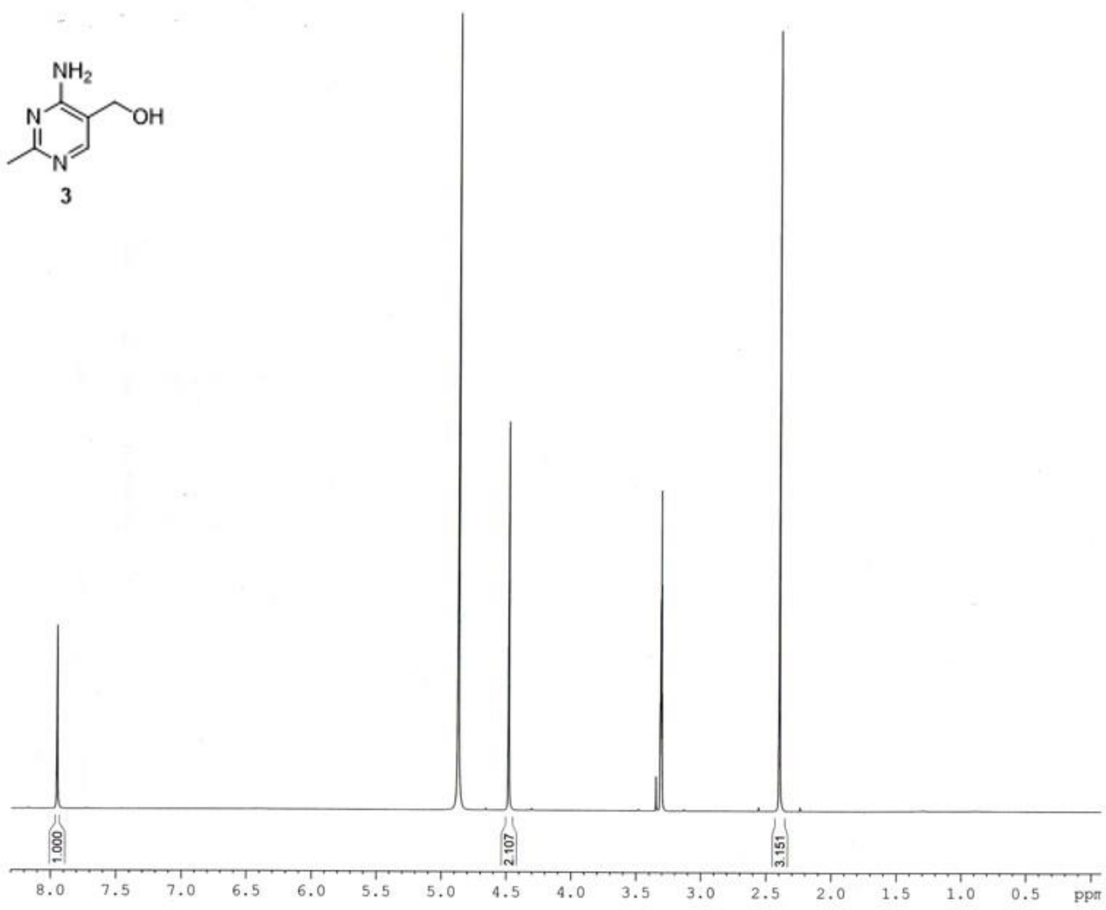

S6 – ^{13}C NMR spectrum of *(4-amino-2-methylpyrimidin-5-yl)methanol (3)*

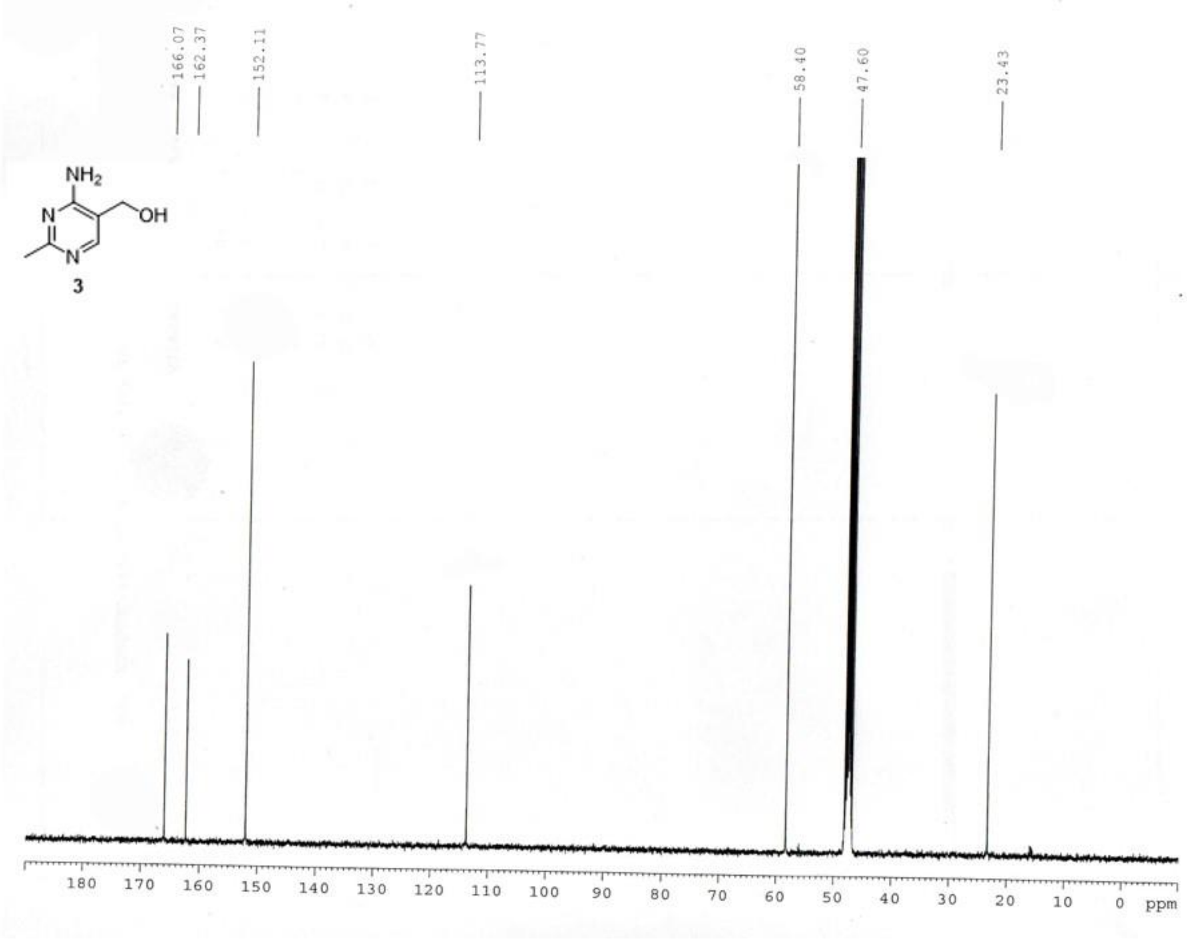

S7 – ^{1}H NMR spectrum of *2'-methylthiamine*

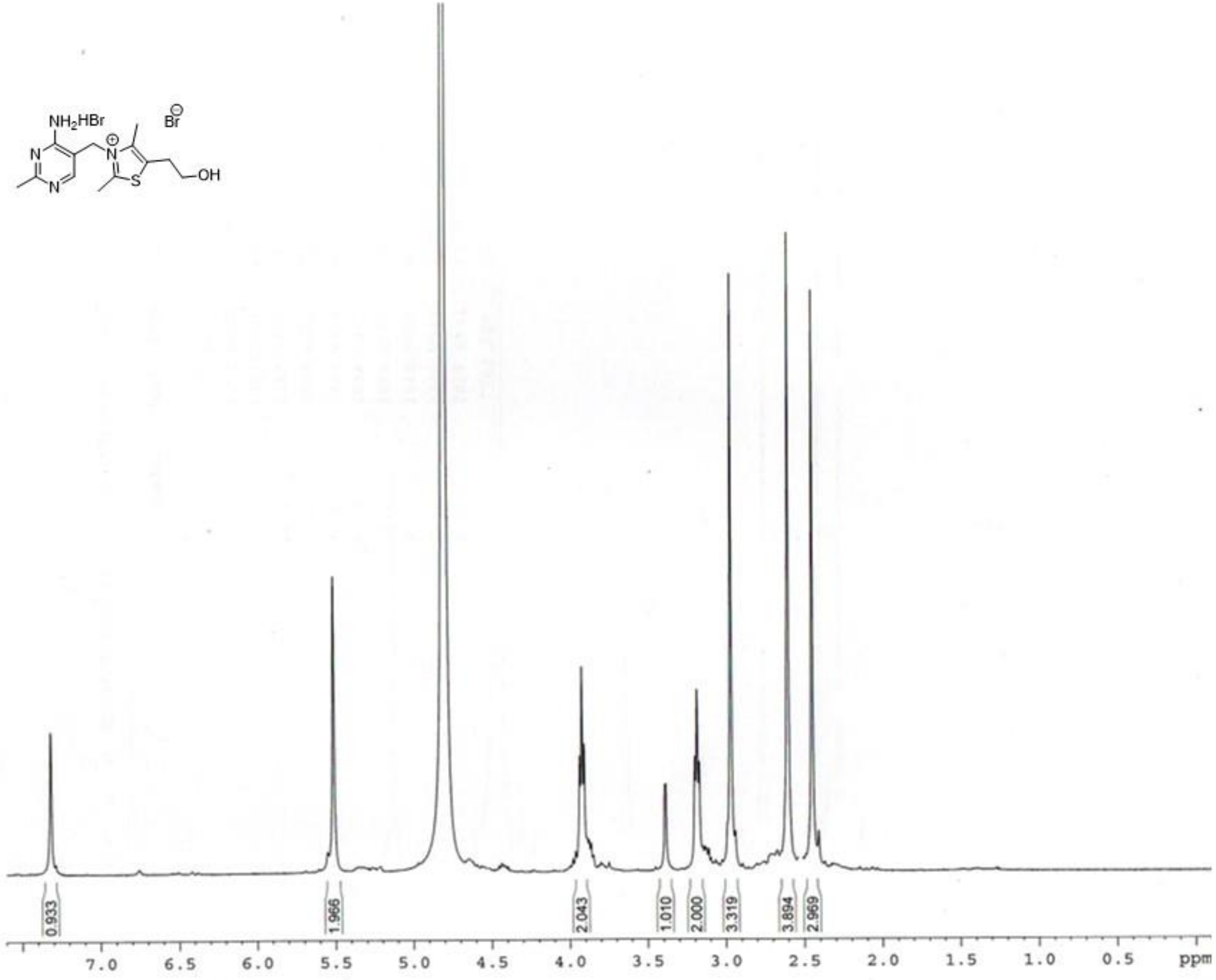

S8 – ^{13}C NMR spectrum of *2'-methylthiamine*

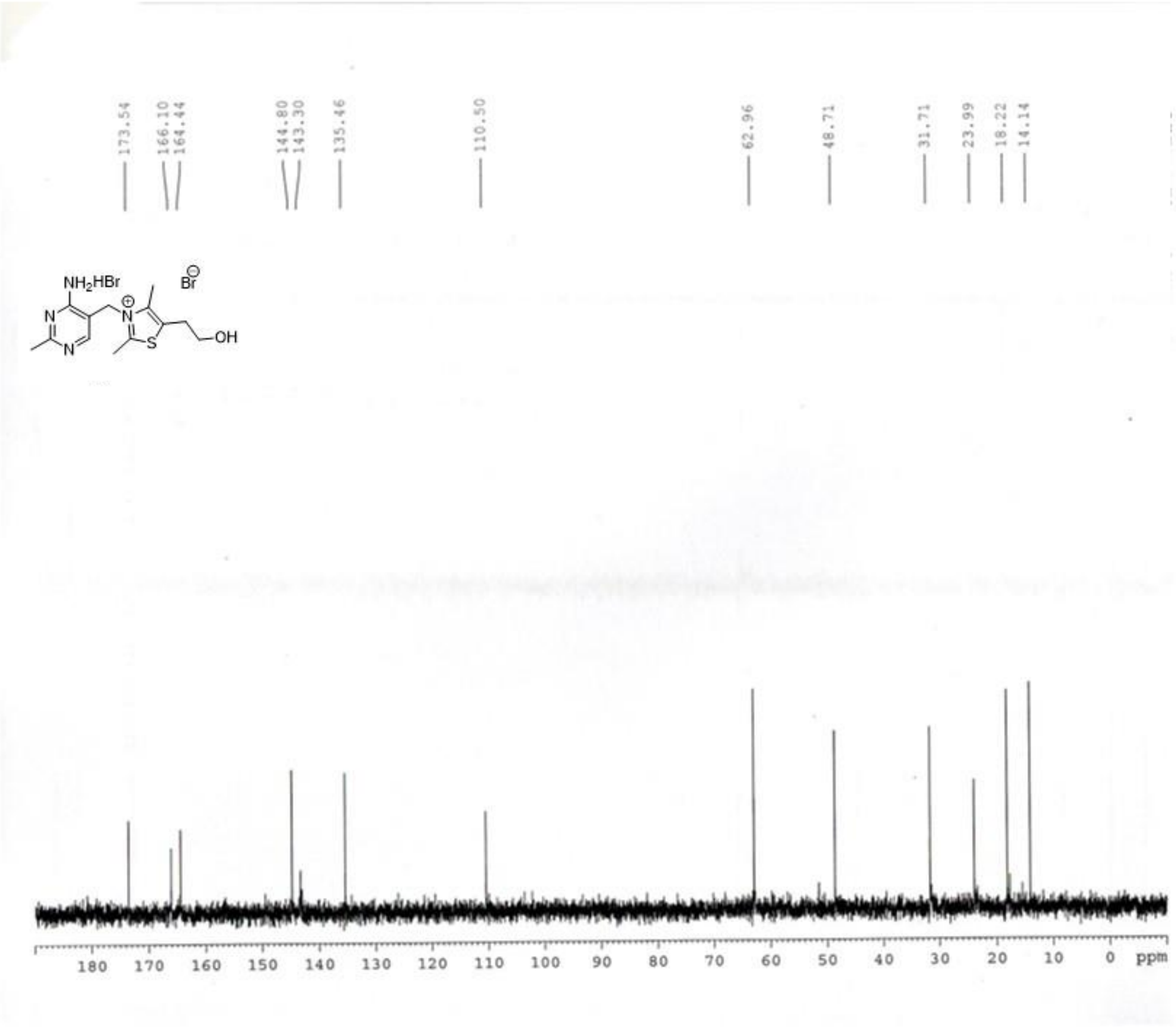

S9 – HPLC chromatogram of *2'-methylthiamine*

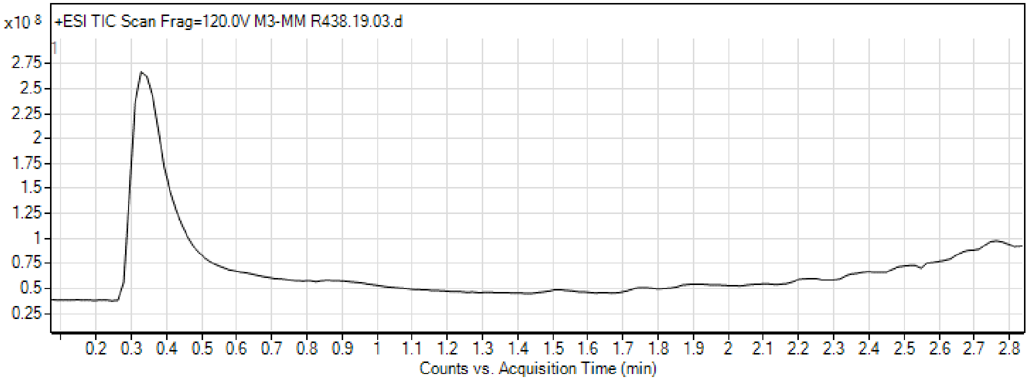

S10 – ESI-HRMS of *2'-methylthiamine*

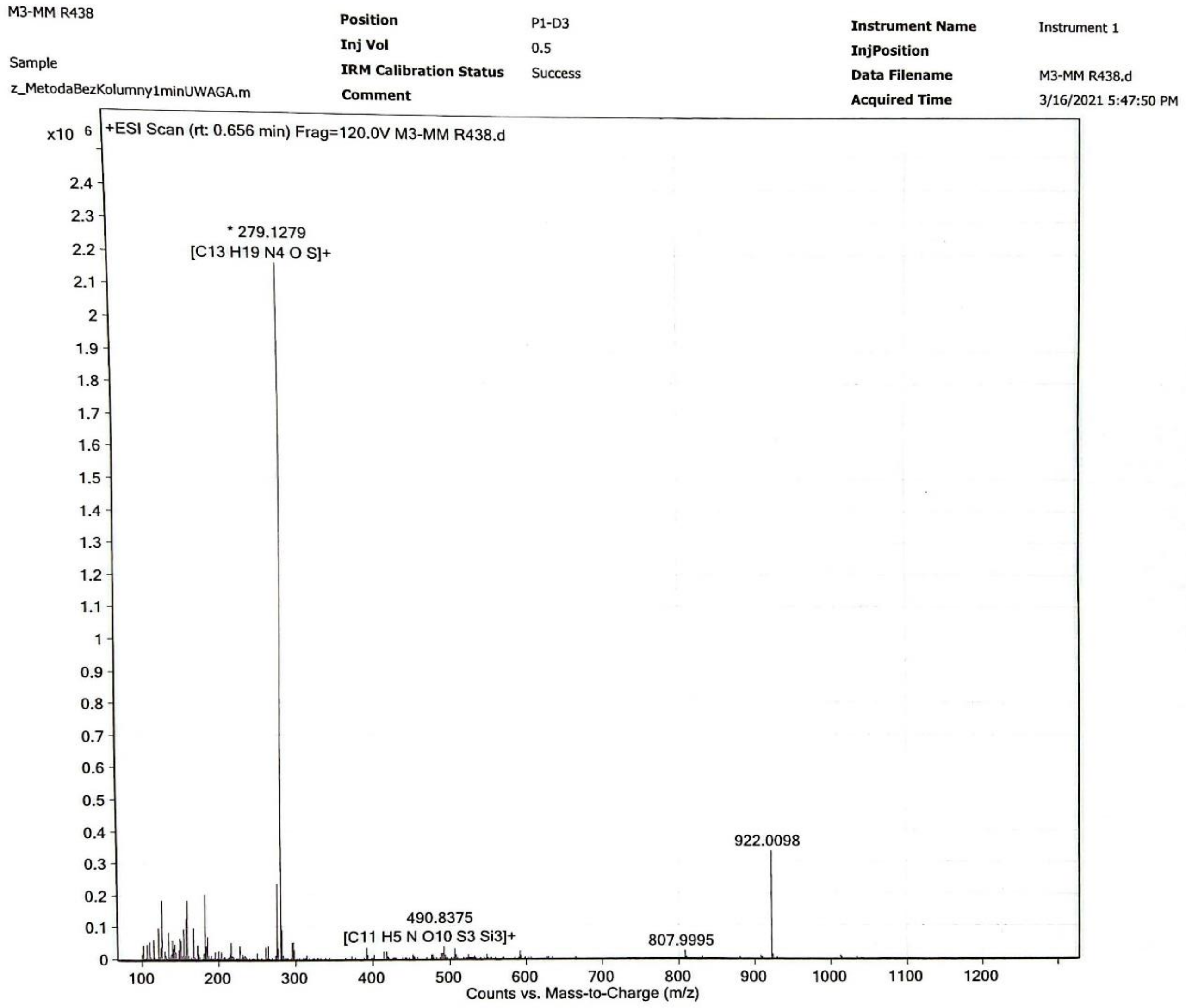

Table S1. Statistical parameters of 100-ns molecular dynamics simulation of OCT1 and its complexes. APO – unliganded form, TIA , MTA, OXT– complexes with thiamine, 2-methylthiamine and oxythiamine. Average - arithmetic mean, SD - standard deviation, SD/average - coefficient of variation.

RMSD

	APO	TIA	MTA	OXT
AVE	2.52	3.03	2.62	3.02
SD	0.36	0.37	0.48	0.67
SD/ave	0.14	0.12	0.18	0.22

SASA

	APO	TIA	MTA	OXT
AVE	26229.05	26148.81	26316.52	26072.81
SD	365.33	472.01	440.67	315.60
SD/ave	0.01	0.02	0.02	0.01

RG

	APO	TIA	MTA	OXT
AVE	27.89	27.61	27.94	27.45
SD	0.21	0.19	0.22	0.26
SD/ave	0.01	0.01	0.01	0.01

RMSF

	APO	TIA	MTA	OXT
AVE	1.45	1.44	1.25	1.51
SD	0.74	0.80	0.75	1.06
SD/ave	0.51	0.56	0.60	0.70
